# Assessing the representativeness of trials of Sodium-glucose Cotransporter-2 inhibitors in type 2 diabetes: a comparison of individual-level trial data and people newly prescribed treatment in a Welsh routine care database

**DOI:** 10.1186/s12916-025-04492-2

**Published:** 2025-11-26

**Authors:** Peter Hanlon, Heather Wightman, Michael Sullivan, Jennifer S. Lees, Elaine W. Butterly, Lili Wei, Ryan McChrystal, Eva Whalley, Saleh Ali Almazam, Khalid Alsallumi, John Petrie, Amanda Adler, Naveed Sattar, Daniel R. Morales, Bruce Guthrie, David McAllister

**Affiliations:** 1 University of Glasgow School of Health and Wellbeing, Clarice Pears Building, 90 Byres Road, Glasgow, UK; 2 University of Glasgow School of Cardiovascular and Metabolic Health, Glasgow, UK; 3 Pharmacology and Toxicology Department, College of Pharmacy, Umm Al-Qura University, Makkah, Saudi Arabia; 4 Pharmaceutical Practices Department, College of Pharmacy, Umm Al-Qura University, Makkah, Saudi Arabia; 5 Diabetes Trials Unit, University of Oxford, Oxford, UK; 6 Population Health and Genomics, School of Medicine, University of Dundee, Dundee, UK; 7 Usher Institute, University of Edinburgh, Edinburgh, UK

**Keywords:** Type 2 diabetes, Sodium Glucose Co-transporter 2 inhibitors, Randomised controlled trials, Applicability, Representativeness, Adverse events, Comorbidity, Multimorbidity

## Abstract

**Background:**

Randomised controlled trials are often criticised for excluding people with multiple long-term conditions. This study used individual participant data for 25 trials of sodium glucose co-transporter-2 inhibitors (SGLT2i) to compare baseline characteristics, comorbidities, and event rates between trial participants and community SGLT2i-treated people in routine care.

**Methods:**

Trials were identified through systematic review with subsequent application for individual-level data. Community SGLT2i-treated people in routine care were identified from the Secure Anonymised Information Linkage (SAIL) databank (Wales, UK). For each trial, we applied the eligibility criteria to the community SGLT2i-treated populations. We then (i) assessed the proportion eligible/ineligible for each trial, (ii) compared age, sex and number of comorbidities between trial participants and those eligible/ineligible in routine care, (iii) compared rates of serious adverse events in the trials to the expected rate in community SGLT2i-treated participants and (iv) compared the rate of major adverse cardiovascular events (MACE), all-cause mortality, non-cardiovascular mortality, and estimated glomerular filtration rate (eGFR) slope between trial and community participants.

**Results:**

The number of comorbidities was consistently lower in trial populations compared to community SGLT2i-treated who met trial eligibility criteria. Compared with other trial populations, in the large cardiovascular outcome trials (CANVAS, CANVAS-R, CREDENCE and EMPA-REG) levels of participant comorbidity were higher; comorbidity differences between trial and community were smaller; and serious adverse event rates were broadly similar to the expected rate based on the community. For the remaining trials, the serious adverse event rate was lower in the trials than the expected rate based on community SGLT2i-treated participants. In the cardiovascular outcome trials, rates of MACE, mortality and decline in eGFR slope were similar or higher in trial populations.

**Conclusions:**

While people with comorbidity are under-represented in most trials compared to a Welsh routine care population, the large cardiovascular outcome trials are more representative of SGLT2i-treated patients and have similar rates of serious adverse events. Therefore, while our findings support calls for caution regarding trial representativeness, the criticism that trials are not representative does not apply equally to all trials. Our results broadly support the applicability of cardiovascular outcome trials to people currently treated with SGLT2i within routine clinical practice.

**Supplementary Information:**

The online version contains supplementary material available at 10.1186/s12916-025-04492-2.

## Background

Drugs such as Sodium-glucose Cotransporter-2 inhibitors (SGLT2i) are an important advance in the management of type 2 diabetes [1]. In addition to improving glycaemia, randomised controlled trial (RCT) evidence shows that SGLT2i reduce the risk of both cardiovascular events and decline in kidney function [2–5]. RCT evidence provides the most internally valid estimate of the efficacy of pharmacological agents, but the applicability of trial findings to people in routine care can be less certain [6–10]. There are concerns that the participants recruited to RCTs are often poorly representative of the populations who receive treatment in routine care [8, 11, 12]. Specifically, people with multiple long-term conditions are often under-represented in RCTs [9], potentially threatening the applicability of their findings. Comorbidity (the presence of a long-term condition in the presence of an index condition) is almost ubiquitous among people with type 2 diabetes and is associated with adverse outcomes such as mortality and hospitalisation [13, 14]. People with comorbidities may be excluded from RCTs through explicit exclusion criteria (which are not always well justified [6]) or because the process of recruitment, screening and monitoring may act as a barrier to participation of people with multiple conditions [15]. Therefore, it is important to examine the representation of people with comorbidities in RCTs for treatments like SGLT2i, because comorbidity is the norm within the target population.

Assessing the representativeness of RCTs can be challenging, and there are various approaches. The commonest approach is to apply trial eligibility criteria to real-world data, and estimate the percentage who would in theory be eligible. However, this approach is not very informative as to how trial participants and real-world patients differ. Direct comparisons of baseline characteristics of actual trial participants to people in routine care are arguably more informative, especially where individual-participant data can be obtained [8] and therefore seldom-reported trial participant characteristics such as comorbidity can be compared [9]. Recently, we have also proposed assessing the rate of serious adverse events within a trial, and comparing these to the expected rates of similar events within routine care [11]. Any event within a trial context that results in hospitalisation or death is considered a serious adverse event, regardless of whether it is thought to be related to the trial treatment [16]. As such, if a trial population is representative of the target population in terms of health status, one would expect the rate of serious adverse events within the trial to be similar to the rate of hospitalisations and deaths among people eligible for treatment within routine care.

This study sought to combine these approaches to assess the representativeness of trials of SGLT2i for type 2 diabetes, comprehensively. Using a set of trials for which we have obtained individual participant data, we aimed to apply each trial’s eligibility criteria to people treated in the community and then compare age, sex, number of comorbidities and rates of adverse health outcomes between people included in randomised controlled trials and people treated with SGLT2i in the community who would have been eligible for each trial.

## Methods

### Overview

**Fig. 1:**
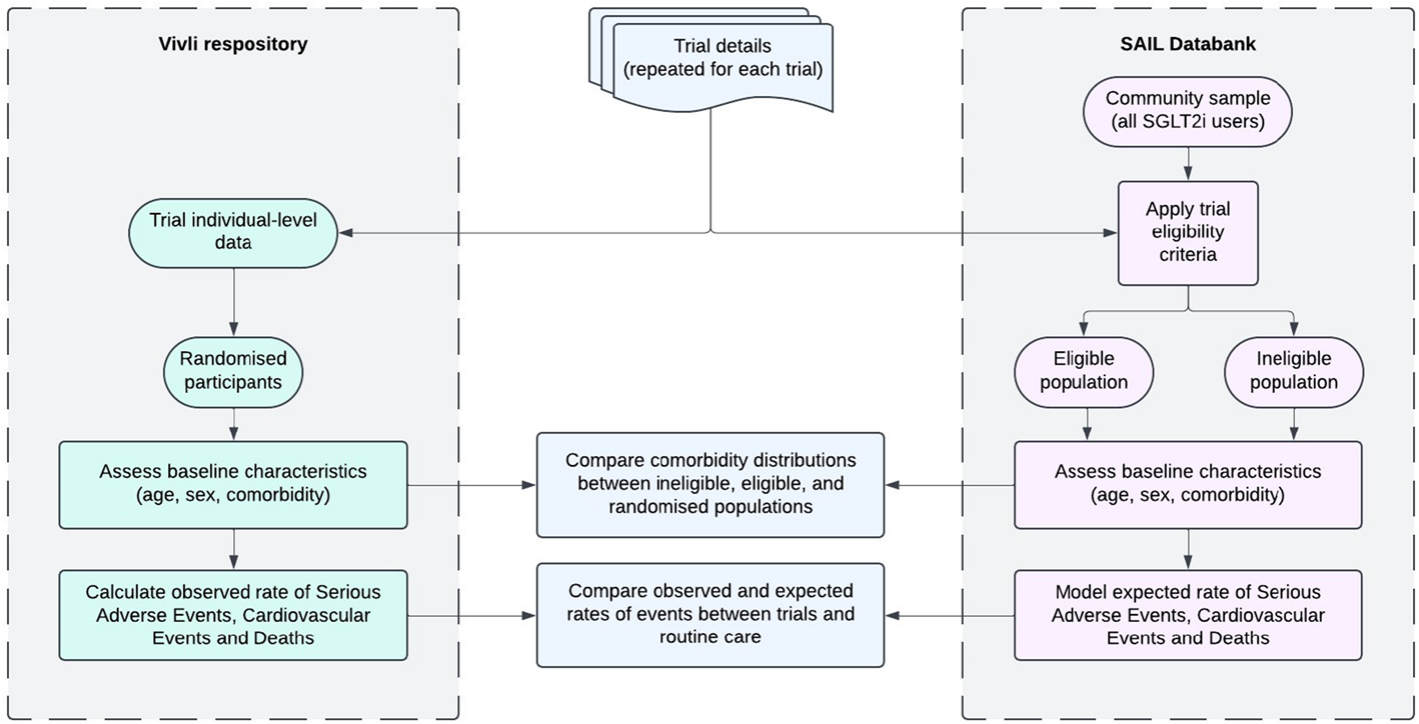
Overview of analysis process

The approach to analysis is summarised in Fig. 1. We sought to compare characteristics, comorbidity counts, and rates of events (conditional on comorbidity) between three distinct groups:

- Participants in trials of SGLT2i for type 2 diabetes (“trial participants”)
- Community SGLT2i-treated people who meet trial eligibility criteria
- Community SGLT2i-treated people who do not meet trial eligibility criteria

### Data sources

#### Trial data

We identified trials of SGLT2i for type 2 diabetes through a systematic review of trials for glucose-lowering agents (reported elsewhere) [17]. Briefly, the systematic review inclusion criteria were phase 3 or 4 randomised controlled trials conducted in adults with type 2 diabetes. Trials within the review were eligible if they compared SGLT2i, glucagon-like peptide-1 receptor agonists or dipeptidyl peptidase 4 inhibitors to placebo or active comparator, and assessed HbA1c, body weight and/or major adverse cardiovascular events. From included trials, we selected SGLT2i trials and sought access to individual participant data available through the Vivli repository. We excluded trials in which medical history data (required to assess comorbidities) were not collected or were redacted at the level of MedDRA preferred terms.

#### Community comparison

For the comparator population of people using SGLT2i in the community, we accessed data from the Secure Anonymized Information Linkage (SAIL) Databank. Briefly, SAIL is a database of routinely collected healthcare data including coded primary care data (including prescriptions, diagnoses and test results) with linked hospital inpatient and mortality data [18]. Patient data were included in SAIL if the patient is registered with a participating primary care practice. Approximately 70% of the population of Wales is covered, and the sample is nationally representative in terms of age, sex and socioeconomic position [9].

We identified all individuals with type 2 diabetes who had been prescribed an SGLT2i prior to 1st November 2024. We excluded individuals who had joined the database less than a year before the first recorded prescription of a SGLT2i to ensure we were assessing incident use and to improve the ascertainment of long-term conditions [19].

### Measures

#### Multiple long-term conditions

Within the trial data and within the community comparison, we quantified the number of long-term conditions in addition to type 2 diabetes. We selected conditions based on a previously published Delphi consensus paper on measuring multimorbidity in health research [20]. We included all conditions apart from chronic Lyme disease and recurrent urinary tract infections (as we were unable to determine chronicity from the available data). This resulted in a list of 57 long-term conditions.

To identify conditions in the trial data, we manually mapped each of these long-term conditions to preferred terms within the MedDRA classification. We then applied this list of terms to the baseline medical history data within the individual participant data for each trial. Chronic kidney disease (CKD) was identified using eGFR criteria rather than MedDRA code. eGFR was calculated based on the single, most recent creatinine value using the CKDEpi equation [21]. CKD was identified as baseline eGFR < 60 mL/min/1.73m^2^.

In the community SGLT2i-treated population, we identified these same long-term conditions using Read version 2 codes (diagnostic codes used within primary care data in SAIL databank) and ICD-10 codes (used in linked hospital data for participants who had been admitted to hospital) [22]. Code lists for each of the included conditions were based on the CALIBER code lists where available (see https://phenotypes.healthdatagateway.org/) and, where this was unavailable, on previously published code lists. Conditions were considered present when any relevant diagnostic code had been recorded in either primary or secondary care data prior to the first recorded date of SGLT2i prescription. As in the trial data, CKD was identified using eGFR rather than Read codes.

For each participant within each data source, we calculated (i) the total number of comorbidities (not including type 2 diabetes), (ii) the total number of cardiometabolic comorbidities (comprising stroke, coronary artery disease, heart failure, peripheral artery disease, heart valve disorders, arrhythmia, venous thromboembolic disease, aneurysm, hypertension, and chronic kidney disease) and (iii) the total number of non-cardiometabolic comorbidities (comprising all other conditions).

#### Trial eligibility criteria

Within the community SGLT2i-treated population, we implemented the eligibility criteria for each trial to identify those who would have been eligible or ineligible for each trial at the time of first SGLT2i prescription.

Inclusion criteria were gathered from clincialtrials.gov, published trial protocols, and published results papers for each trial.

Within the community SGLT2i-treated population, each of these criteria were implemented using data prior to the initial SGLT2i prescription. Age and sex were based on demographic data held within SAIL databank primary care records. Eligibility criteria based on comorbidities were implemented using Read codes and ICD-10 codes from linked primary and secondary care data, respectively. Criteria based on specific values (e.g. HbA1c, systolic blood pressure) were applied to coded values within primary care data, taking the most recent value prior to initial prescription (limited to a 2-year lookback). As in the trial data, eGFR was calculated from the single, most recent creatinine value using the CKDEpi equation [21]. Eligibility criteria based on procedures (e.g. no bariatric surgery within the last 2 years) were identified from procedure codes from linked hospital inpatient data. We did not implement any eligibility criteria based on ethnicity as these data are incomplete within SAIL.

### Outcomes

For analysis of outcomes, trial participants were restricted to those randomised to SGLT2i, and compared with community SGLT2i-treated participants who met trial eligibility criteria.

#### Serious adverse events

In randomised controlled trials, serious adverse events are defined as events that result in death, hospital admission, are life threatening, result in disability or result in a birth defect. Within the trial data, we identified incident serious adverse events and calculated time at risk for each individual. Within the community SGLT2i-treated population, we identified incident all-cause hospitalizations or deaths (which, by definition, would be serious adverse events in a trial context). For each trial, we identified events occurring after randomisation and before the primary endpoint. We then applied this same time-window of observation to the comparator community SGLT2i-treated population for each trial.

#### Deaths

In both trial and community samples, we identified all recorded deaths and then further classified these into cardiovascular and non-cardiovascular deaths. In the trial data, deaths adjudicated as being cardiovascular deaths with respect to the MACE endpoint of the trial were classified as cardiovascular deaths, and the rest as non-cardiovascular deaths. In the community sample, cardiovascular death was defined from national mortality registration data as those where the underlying cause of death was an ICD-10 code starting with “I”, and non-cardiovascular death was defined as all other deaths.

#### Major adverse cardiovascular events

Within the trial data, we defined 3-point major adverse cardiovascular event (MACE) as the first event of non-fatal myocardial infarction, non-fatal ischaemic stroke, or cardiovascular death. Within the community SGLT2i-treated population, we identified similar events by identifying ICD-10 codes from linked hospital episode statistics for myocardial infarction and ischaemic stroke, and cardiovascular death from linked mortality registration records.

#### Estimated glomerular filtration rate slope

We calculated total eGFR slope in each population using a mixed effects model with an unstructured residual variance–covariance matrix using code developed by the SGLT2 inhibitor Meta-Analysis Cardio-Renal Trialists Consortium (SMART-C) [23].

In the trial populations, total eGFR slope was calculated as the annualised rate of change of eGFR from baseline, using all available eGFR values during the follow-up period of the trial until the end of follow-up. We restricted the analysis to trials with at least 2 years of follow-up, as shorter time frames have not been validated to predict future risk of kidney failure [24, 25], and there were insufficient measurements in the corresponding community sample to calculate slope accurately over time periods shorter than 2 years.

In the community SGLT2i-treated population, total eGFR slope was calculated as the annualised rate of change of eGFR from baseline (the most recent value prior to initiation of SGLT2i), using all available eGFR values during the timeframe of the corresponding trial, and for a minimum of 2 years.

We calculated total eGFR slope (rather than chronic slope), as eGFR is not routinely tested in the period immediately after treatment initiation within the community SGLT2i-treated population (in keeping with current clinical guidance), precluding accurate calculation of the chronic slope. The spline term for the acute effect of SGLT2i was set at 21 days following initiation (corresponding to the first post-treatment sample within the trials).

### Statistical analysis

#### Descriptive statistics

For each of the included trials, we generated descriptive statistics (counts and percentage, or mean and standard deviation) for age, sex and each comorbidity count among (i) community SGLT2i-treated participants who were ineligible for the trial, (ii) community SGLT2i-treated participants who were eligible for inclusion and (iii) trial participants who were included and randomised.

#### Distribution of comorbidities

We summarised the count of total comorbidities, cardiometabolic comorbidities and non-cardiometabolic comorbidities within each population (ineligible, eligible and included) using statistical distributions appropriate to count data (e.g. Poisson or negative binomial). Fit of each distribution was assessed visually (plotting the fitted distribution over the observed counts) and using Kolmogorov-Smirnoff tests. We selected the best-fitting distribution for each population and each trial and exported the parameters estimates from the secure analysis platform. This allowed us to plot the distributions from each population together, while the individual-level data remained within their respective secure analysis platforms.

#### Observed and expected event rates

For each trial, we compared the observed to expected SAE ratio. The community rates for each outcome (serious adverse events, MACE and death), separately, were obtained by fitting Poisson or negative binomial regression models on age and sex as well as (for model 2) comorbidity count. We included an offset term for time at risk which was calculated separately for each outcome as the first of time to first event, de-registering from a participating practice (and thus no longer being observable) or the end of the follow-up period of the corresponding trial (whichever occurred first). Non-linear associations for age and comorbidity count were accommodated using up to two fractional polynomial terms. We assessed interaction terms between covariates and included these where they improved model fit, which we assessed using likelihood ratio tests and comparing AIC. We then exported the model coefficients (\beta) and variance–covariance matrices (\sigma) from the secure analysis platform to allow them to be applied to the trial data (which was held separately).

We then used the coefficients to estimate the expected event rates for each trial given the trial-distribution of age, sex distribution and (for model 2) comorbidity count. These expected rates, number of participants and trial duration were then used to estimate the expected counts. We then calculated the SAE ratio as the observed/expected counts.

We calculated 95% confidence intervals reflecting uncertainty in both the expected counts and the observed counts. We allowed for uncertainty in the expected counts by repeating these analyses using 10,000 samples representing the uncertainty in the regression model coefficients. These were obtained by sampling from a multivariate normal distribution (mean = coefficient point estimate, sigma = variance–covariance matrix). These coefficient samples were then applied to the trial covariate distribution to obtain a set of 10,000 expected counts for each trial. We allowed for uncertainty in the observed counts by obtaining 10,000 samples from a beta distribution (as this is the conjugate prior for the binomial likelihood which is appropriate for proportion data) and multiplying this by the number of participants. For each sample, we calculated the ratio (as above) and obtained a 95% confidence interval as the 2.5th and 97.5th centiles.

## Results

Individual-level data were available for 31 of the 140 trials of SGLT2i included in the systematic review. Six of these were excluded as medical history data were redacted at the level of preferred MedDRA terms. There were 25 trials (*n* = 41,395 participants; range 157 to 7063 per trial) included in the final analysis (out of a total of 140 potentially eligible trials with *n* = 84,230 participants).

There were 29,544 people prescribed SGLT2i within our community sample in whom we assessed trial eligibility. Summary statistics for trial and community SGLT2i-treated populations are shown in Additional File 1, Table S1. Community SGLT2i prescribing rose from 1.5% (2032/137,828) of those with type 2 diabetes in 2015, to 11.4% (20431/178510) in 2024. People prescribed SGLT2i were younger than those who were not prescribed (mean age 58.9 vs 66.8 in 2015) but this difference narrowed by the end of the study period (mean age 64.3 vs 67.6 in 2024). The mean number of comorbidities was lower in those prescribed versus those not prescribed an SGLT2i (2.7 vs 3.2 in 2015); however, this difference also narrowed over time (3.6 vs 3.7 in 2024). The proportion of females was lower among those prescribed (39%) compared to those not prescribed (45%) an SGLT2i.

### Comparison of trial participants and community SGLT2i-treated populations

**Fig. 2:**
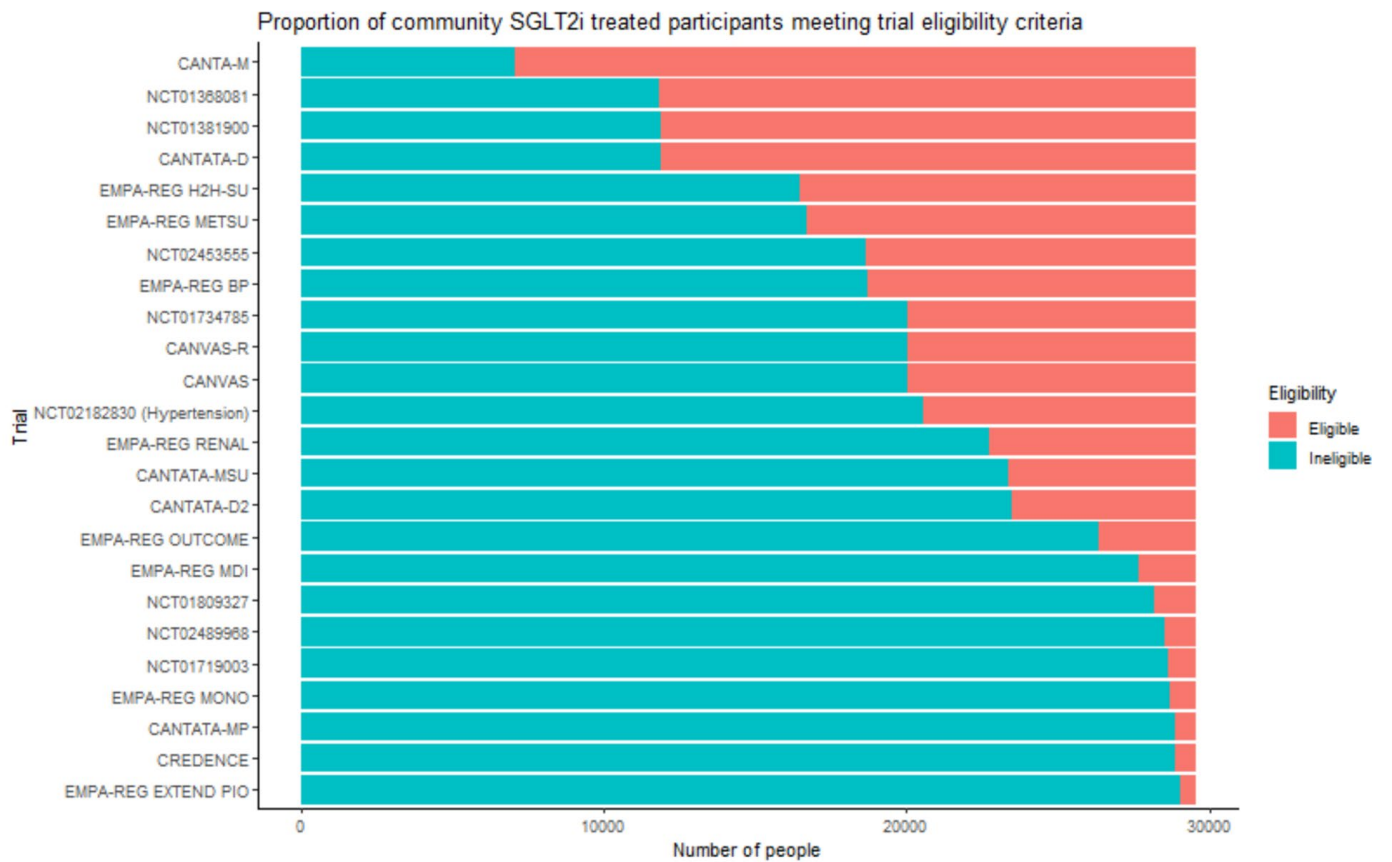
Proportion of community SGLT2i-treated participants meeting trial eligibility criteria. This plot shows the percentage of incident SGLT2 users within SAIL databank who would have met the inclusion criteria for each of the trials

The proportion of community SGLT2i-treated participants who met eligibility criteria for each of the trials is shown in Fig. 2 (range 2% to 76%, median 31%, IQR 4% to 39%). Eighty nine percent of people treated in the community were eligible for at least one of the included trials. Trial participants were often slightly younger than the eligible community SGLT2i-treated participants (Additional File 1, Table S1). In the cardiovascular outcome trials, the percentage of women included was typically low (29 to 37% of trial participants); however, these figures were closely matched by the percentage of women in the community SGLT2i-treated population who were eligible.

**Fig. 3:**
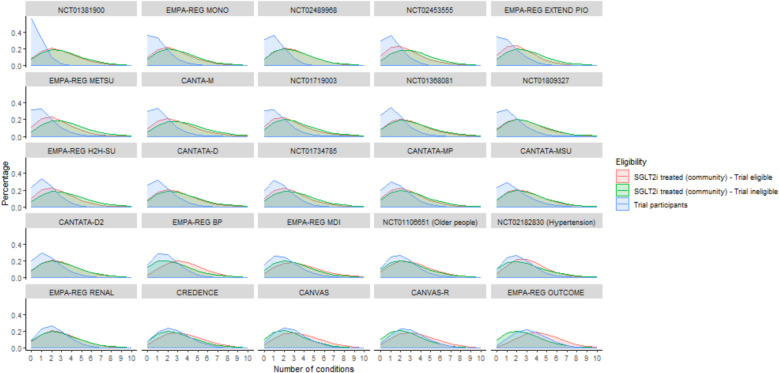
Counts of long-term conditions among trial participants and community SGLT2i-treated eligible/ineligible participants. This plot shows the distribution of comorbidity counts among trial participants (blue), community SGLT2i-treated participants who meet trial eligibility criteria (red) and community SGLT2i-treated participants who did not meet trial eligibility criteria

The distribution of comorbidities within each of the included trials is shown in Fig. 3. When comparing trial participants to eligible community SGLT2i-treated participants, the mean number of comorbidities was lower among participants for all trials. However, this difference was relatively small in the cardiovascular outcome trials and those focusing on higher-risk populations such as older people or those with hypertension or chronic kidney disease. In these trials of higher-risk populations, the mean comorbidity count in the trials was consistently greater than two (ranging 2.2 to 3.4) and around 20% lower than the eligible community SGLT2i-treated participants (ranging 3.1 to 4.1, see Additional File 1, Table S2 showing mean counts and Additional File 1, Table S3 showing the ratio of mean counts between trial participants and community eligible populations). For these trials in “high risk” populations, comorbidity counts in the community SGLT2i-treated ineligible population was lower than in the community SGLT2i-treated eligible (reflecting the selection of higher risk individuals within the trial inclusion criteria). In the remaining trials, the absolute number of comorbidities was lower and the difference in comorbidity counts between trial participants and community SGLT2i-treated populations was greater in magnitude (generally 40 to 60% lower in the trial than in the community). The difference between the treated-eligible and treated-ineligible populations was considerably lower (10–20% lower in the eligible compared to the ineligible), suggesting that while explicit exclusion criteria resulted in some reduction in comorbidity, most of the difference in comorbidity between trial and community SGLT2i-treated populations is not explained by explicit eligibility criteria.

When separating cardiometabolic and non-cardiometabolic comorbidities, trials were more similar to routine care for cardiometabolic comorbidities; however, the differences in non-cardiometabolic comorbidities were more marked (Additional File 1, Table S2 and S3 and Figure S1).

### Rates of serious adverse events

**Fig. 4:**
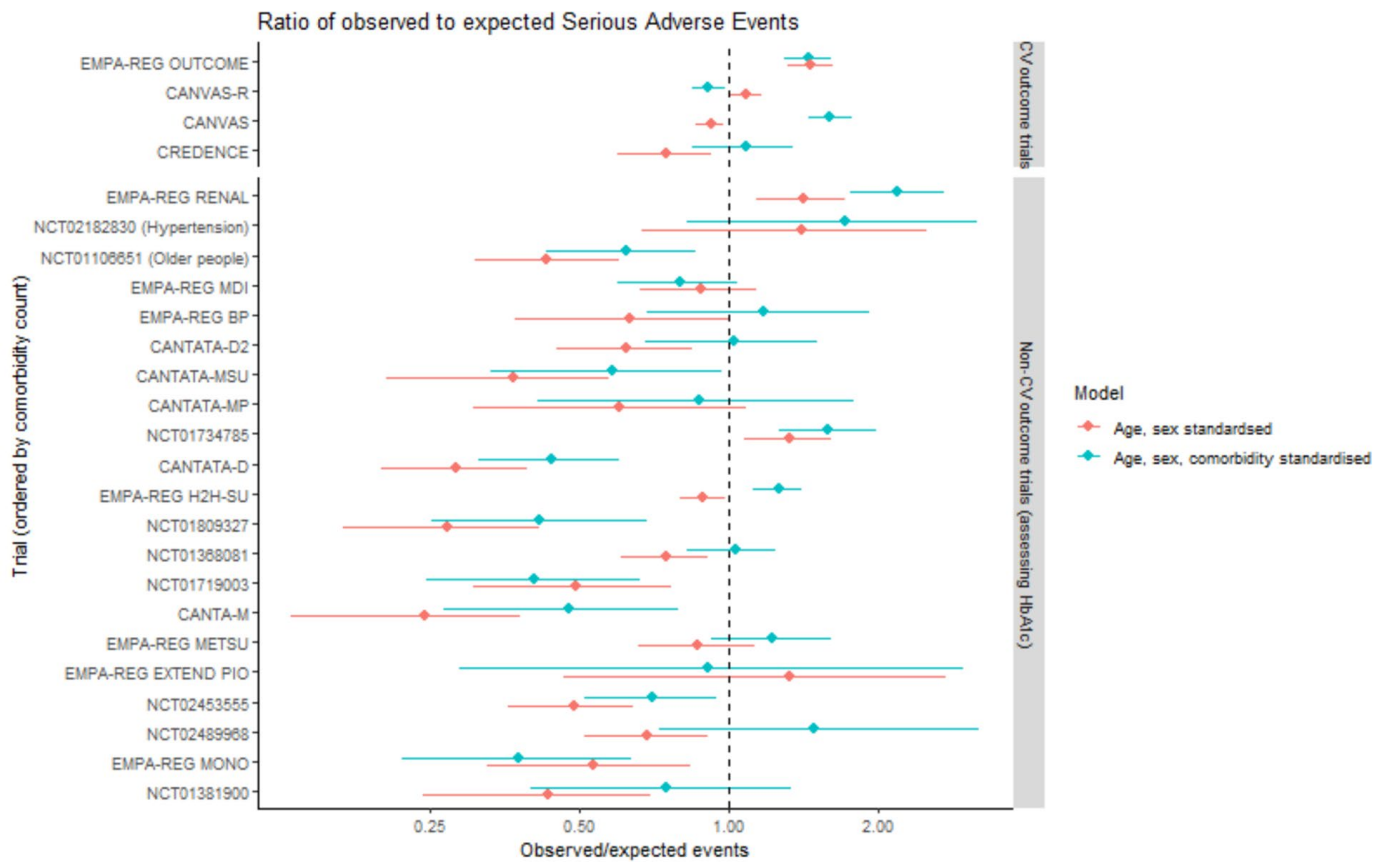
This plot shows the ratio of observed serious adverse events (based on the trial IPD) to the expected number of serious adverse events based on community SGLT2i-treated people who were trial-eligible. Red indicates the analysis standardised to the age-sex distribution of the trial population, blue indicates the analysis standardised to age, sex and comorbidity count. Points show the ratio of observed events (in the trial population) to the expected number of events (based on hospitalisation and deaths among community SGLT2i-treated people meeting trial eligibility criteria). Lines indicate 95% confidence intervals

Figure 4 shows the ratio of observed to expected serious adverse events based on eligible community SGLT2i-treated participants, standardised by age and sex alone (red) and by age, sex and comorbidity (blue). In trials with higher levels of comorbidity, which were also trials that specifically included high-risk populations (based on cardiovascular risk, chronic kidney disease or older age), the ratio of observed to expected serious adverse events was similar or greater to the rate seen in people treated with SGLT2i in routine care. For the remaining trials, the age-sex standardised ratios were < 1, showing that trial participants had significantly lower event rates than community SGLT2i-treated participants (often between half and a quarter of the rate age-sex standardised rate). Differences between trials and routine care were attenuated with additional standardisation by comorbidity count; however, for trials in which the difference was large the difference remained significant after accounting for comorbidity.

### Rates of cardiovascular events, deaths, and change in kidney function

**Fig. 5:**
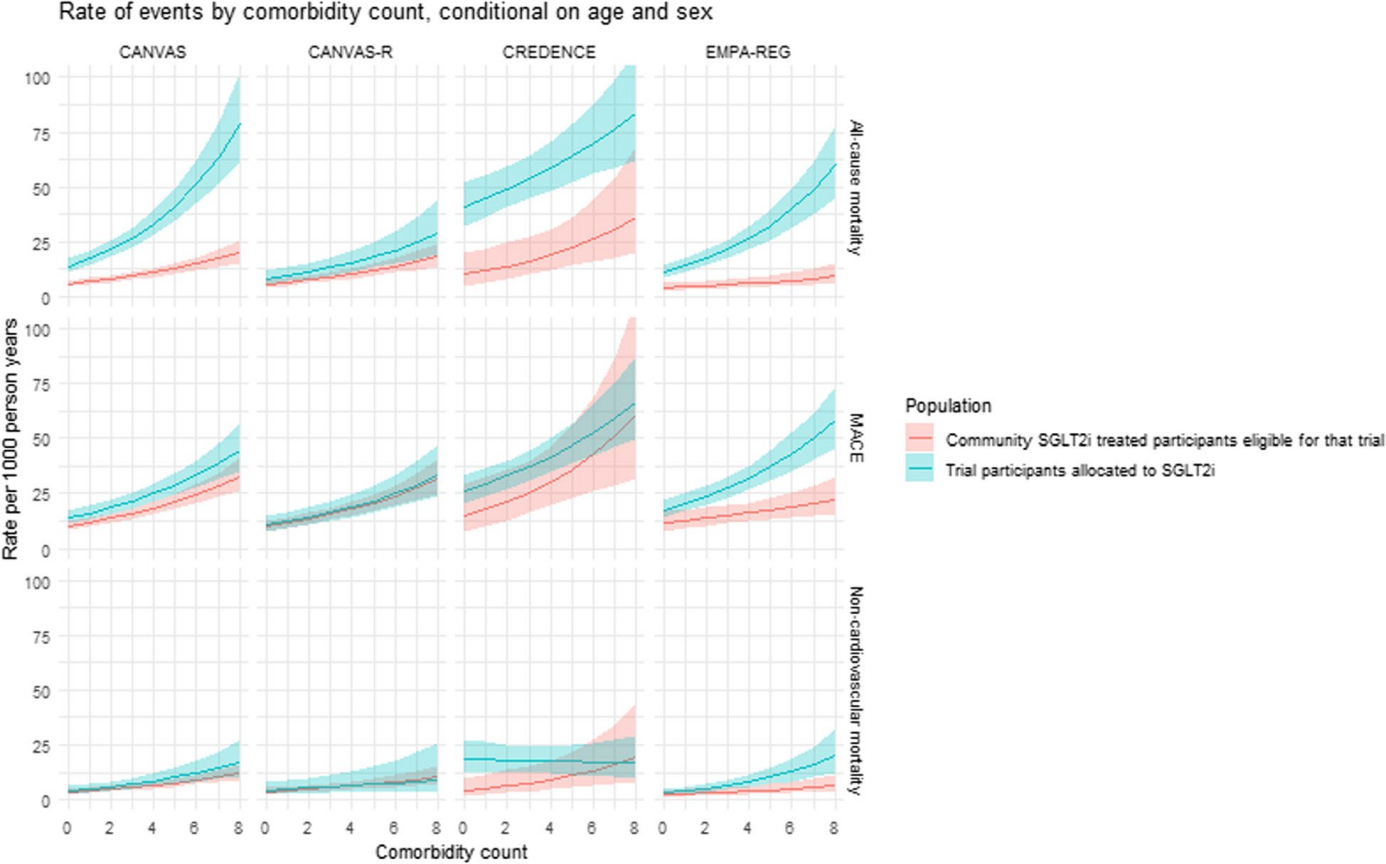
This plot shows the results of a model assessing the rate of all-cause mortality, non-cardiovascular mortality, and major adverse cardiovascular events in trial participants allocated to SGLT2i treatment (blue) and in community SGLT2i-treated people meeting trial eligibility criteria. Rates are estimated across the spectrum of comorbidity counts, at the mean age of each trial, and at the mid-point between estimates for men and women. Lines indicate the estimate while the shaded area shows the 95% confidence interval

**Fig. 6:**
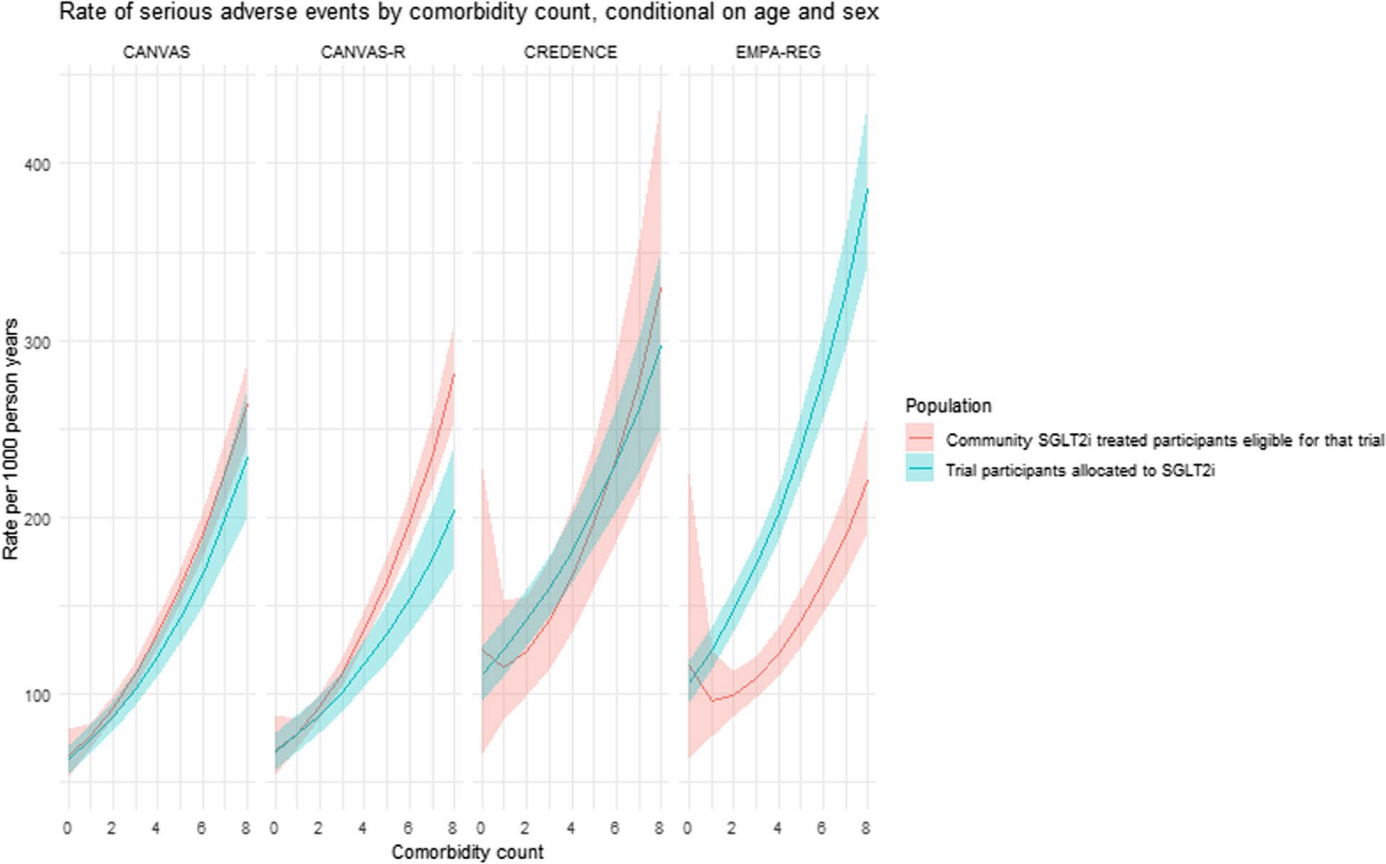
This plot shows the results of a model assessing the rate of serious adverse events in trial participants allocated to SGLT2i treatment (blue) and in community SGLT2i-treated people meeting trial eligibility criteria. Rates are estimated across the spectrum of comorbidity counts, at the mean age of each trial, and at the mid-point between estimates for men and women. Lines indicate the estimate while she shaded area shows the 95% confidence interval

For the four large cardiovascular outcome trials (EMPA-REG, CANVAS, CANVAS-R and CREDENCE) (in which there were sufficient participants and follow-up to model cardiovascular, kidney and mortality outcomes), Fig. 5 shows the rates of serious adverse events, cardiovascular events, all-cause mortality and non-cardiovascular mortality among people on SGLT2i treatment in each trial and community SGLT2i-treated participants who were eligible for the trial. Across all levels of comorbidity, rates of cardiovascular, kidney and mortality outcomes were either comparable or higher in the trial participants compared to community SGLT2i-treated participants eligible for each trial. Figure 6 shows this same comparison for the rate of serious adverse events, which were similar or higher in trial participants than in community SGLT2-treated participants. Finally, the eGFR slope was similar for the treatment arm of each trial and the trial-eligible community SGLT2i-treated participants.

## Discussion

This analysis of individual participant data from 25 trials of SGLT2i in type 2 diabetes showed that trial populations had fewer comorbidities on average than people currently treated with SGLT2i in routine care. However, for the large cardiovascular outcome trials that focused on higher risk populations (often in people with evidence of end-organ damage), trial participants had levels of comorbidity that were closer to those seen among community SGLT2i-treated participants who were eligible for those trials. Furthermore, the rates of adverse clinical outcomes (including target and competing events) were similar or higher in these cardiovascular outcome trials than in people treated in the community who met the inclusion criteria. This suggests that while many trials are unrepresentative, others more closely reflect those currently prescribed SGLT2i in routine care. Given that these large cardiovascular outcome trials are particularly influential in terms of clinical guideline recommendations, this gives some confidence that the promotion of SGLT2i for type 2 diabetes, which is increasingly based on their effects on hard cardiovascular and kidney outcomes rather than explicitly on their glycaemia effects, is appropriate in the context of multiple long-term conditions, at least with respect to people currently being treated.

Our findings should be interpreted with careful consideration of our community comparison population, who were both currently treated and would have met trial eligibility criteria. There are also likely to be individuals who would be eligible for, and may benefit from, SGLT2i treatment but were not prescribed, and therefore are not included in our analysis. Our sample was also drawn from a specific setting (primary care within Wales) and may not necessarily reflect use in other settings or at different time-points. Our assessment of eligibility was made at the point when the SGLT2i was first prescribed, but this may not reflect the point at which this treatment was first indicated (e.g. due to clinical inertia leading to delays between a treatment becoming indicated and it being initiated, particularly earlier in our study period when these treatments were relatively new). As use of SLGT2i becomes more widespread (as is reflected in our findings showing higher comorbidity in more recent, compared to earlier, incident users), some patients are likely to be offered SLGT2i relatively earlier in their disease course, or in the presence of greater degrees of comorbidity than when these drugs were first introduced.

Previous literature showed that a large proportion of people with a given condition (including, but not limited to, type 2 diabetes) do not meet eligibility criteria for most trials [8]. Our findings are consistent with this literature, but also highlight that for many trials the under-representation of people with comorbidity is not fully explained by explicit exclusion criteria. For many trials, including those that appeared most under-representative in terms of comorbidity, we found that comorbidity counts in community SGLT2i-treated participants who were eligible and ineligible for the trial were more similar, despite considerably lower levels in those randomised. When considering these trials with lower comorbidity counts, the significantly lower rates of serious adverse events in trial populations compared to community SGLT2-treated participants who were eligible for those trials also suggest that these differences in comorbidity are likely to reflect genuine differences in the health status of trial participants and people treated in routine care.

While these observations are consistent with the commonly expressed concern that trials are poorly representative of their target populations [26], our findings show that this criticism cannot be levelled equally at all trials. In the context of SGLT2i’s, trials that intentionally recruited high-risk populations (such as those with high cardiovascular risk based on prior events or kidney disease) had levels of comorbidity much closer to those treated in the community, although this was driven by trial participants having higher levels of cardiovascular comorbidity and lower levels of non-cardiovascular comorbidity. Event rates in trial participants were broadly similar or higher to those treated in routine care, including the rate of all-cause serious adverse events and non-cardiovascular mortality. This is an important observation as one source of concern regarding the applicability of trial evidence is that rates of competing risks (such as non-cardiovascular mortality) may be higher in routine care. Our findings suggest this is unlikely to be the case in people currently treated with SGLT2i in routine care. However, as treatment expands to larger numbers of people with type 2 diabetes, this may still be a concern when applying treatment decisions to individuals, particularly people with severe or advanced comorbidities unrelated to diabetes or living with severe frailty, in whom event rates may differ from those included in trials and those currently prescribed SGLT2i treatment in routine care. It would be important to repeat the comparisons we present if and when SGLT2i usage expands.

A challenge when assessing the representativeness of trial populations is selecting the appropriate community sample with whom to compare the trial population. We selected people with type 2 diabetes who were currently prescribed SGLT2i in routine care, as this accurately reflects current real-world use of these agents and allows a more direct comparison than selecting (for example) all people with the index condition. However, a drawback of this approach is that some people may be potentially eligible for treatment in routine care but, for various reasons, may not be prescribed treatment [27]. For example, a study from Denmark demonstrated that people at risk of frailty are less likely to receive treatment with SGLT2i despite being potentially eligible for treatment. This may result in an under-estimation of the difference between trial participants and the target population [28]. Furthermore, as drugs such as SGLT2i become closer to first-line treatments the treated population may diverge from the original trial populations. Repeating similar analyses in future may therefore reveal changes in the representativeness of trial relevant to incident users over time.

Strengths of our analysis include the use of individual participant data, study selection nested within a large systematic review and the application of multiple different analyses to assess representativeness. There are also important limitations. First, it was not possible to implement every trial eligibility criterion within the routine data because some characteristics (e.g. ethnicity) could not be identified, and others are implicit (such as investigator discretion). Second, while our list of comorbidities was based on published consensus, the coding system in which these were operationalised (Read codes versus MedDRA codes) were different, which could lead to differences in the quantification of some comorbidities between data sources. Third, while hospitalisations and deaths make up the majority of serious adverse events in trials, some other events (such as those resulting in disability) also qualify. This could result in an over-estimation of the rate in trials compared to routine care (in which only hospitalisations and deaths were quantifiable). Fourth, while we implemented similar definitions of MACE between trials and routine care, these were based on hospitalisation and death codes in routine care and on adjudicated events within the trials, which could introduce some discrepancies in measurement. Fifth, while our community sample was nationally representative of people in Wales prescribed SGLT2i, this may not precisely reflect comorbidity, hospitalisation rates, or SGLT2i usage in other settings. Management of type 2 diabetes in the UK is strongly influenced by National Institute of Health and Care Excellence guidance. Comparisons between trial and routine care populations receiving treatment may therefore be different in other settings where the culture, incentives and controls around who receives treatment are different. Sixth, while restricting our analysis of the routine care data to people who received SGLT2i ensured that the comparator population were “eligible for treatment in routine care”, this may have resulted in the exclusion of people who either declined treatment or who clinicians were hesitant to treat despite being technically eligible (e.g. according to guidelines). Seventh, the trials to which we had access to IPD were not a random sample of SGLT2i trials and did not include trials of some agents within this class (e.g. dapagliflozin). As we found that representativeness of the included trials was highly variable, our findings should not be interpreted as an assessment of the whole body of evidence for SGLT2i. Finally, as the use of these agents becomes more widespread, treatment is likely to extend to populations who are likely to be more different to trial participants, and who may have rather different patterns of cardiovascular events and adverse events. There is a need for further research to understand the representativeness of trials in people not currently treated with SGLT2i as treatment expands.

## Conclusions

Trials of SGLT2i for type 2 diabetes varied considerably in their representativeness, across multiple metrics, with respect to people in Wales who were prescribed SGLT2i in routine care. While many glycaemia efficacy trials included healthier and less comorbid populations than those treated in routine care, participants in large cardiovascular outcome trials appeared to be largely comparable to people in routine care who received treatment in terms of comorbidity. These findings provide a degree of reassurance to decision-makers uncertain as to the applicability of these trials to patients in real-world settings, such as people with multiple long-term conditions.

## Supplementary Information

Additional file 1: Table S1. Age and sex characteristics for community SGLT2i treated individuals who meet eligibility criteria for each trial, those who did not meet eligibility criteria, and actual trial participants. Table S2. Mean (sd) number of comorbidities among community SGLT2i treated individuals who meet eligibility criteria for each trial, those who did not meet eligibility criteria, and actual trial participants. Table S3. Ratio of comorbidity counts comparing (1) trial participants and community SGLT2i treated people who were eligible for the trial, and (2) community SGLT2i treated people who were eligible for the trial and community SGLT2i treated people who were not eligible for the trial. Figure S1. Distribution of total, cardiometabolic and non-cardiometabolic comorbidities in trials and in routine care.

## Data Availability

Trial IPD are available upon application to the trial sponsors via Vivli https://vivli.org/. SAIL data are available upon application to the SAIL Information Governance Review Panel (IGRP) https://saildatabank.com/sail/apply-to-work-with-the-data/.

## Abbreviations

eGFR: Estimated glomerular filtration rate
IPD: Individual participant data
MACE: Major adverse cardiovascular event
SAE: Serious adverse event
SGLT2i: Sodium Glucose Co-transporter 2 inhibitor

## References

[1] Type 2 Diabetes in Adults: Management (NICE Guideline 28) https://www.nice.org.uk/guidance/ng28. Accessed Sep 2025.

[2] Shi Q, Nong K, Vandvik PO, Guyatt GH, Schnell O, Ryden L, et al. Benefits and harms of drug treatment for type 2 diabetes: systematic review and network meta-analysis of randomised controlled trials. BMJ. 2023;381:e074068. 37024129 10.1136/bmj-2022-074068 PMC10077111

[3] Zinman B, Wanner C, Lachin JM, Fitchett D, Bluhmki E, Hantel S, et al. Empagliflozin, cardiovascular outcomes, and mortality in type 2 diabetes. N Engl J Med. 2015;373(22):2117–28. 26378978 10.1056/NEJMoa1504720

[4] Neal B, Perkovic V, Mahaffey KW, De Zeeuw D, Fulcher G, Erondu N, et al. Canagliflozin and cardiovascular and renal events in type 2 diabetes. N Engl J Med. 2017;377(7):644–57. 28605608 10.1056/NEJMoa1611925

[5] Perkovic V, Jardine MJ, Neal B, Bompoint S, Heerspink HJL, Charytan DM, et al. Canagliflozin and renal outcomes in type 2 diabetes and nephropathy. N Engl J Med. 2019;380(24):2295–306. 30990260 10.1056/NEJMoa1811744

[6] Van Spall HG, Toren A, Kiss A, Fowler RA. Eligibility criteria of randomized controlled trials published in high-impact general medical journals: a systematic sampling review. JAMA. 2007;297(11):1233–40. 17374817 10.1001/jama.297.11.1233

[7] Angus DC, Huang AJ, Lewis RJ, Abernethy AP, Califf RM, Landray M, et al. The integration of clinical trials with the practice of medicine: repairing a house divided. JAMA. 2024;332(2):153–62. 38829654 10.1001/jama.2024.4088 PMC12045079

[8] He J, Morales DR, Guthrie B. Exclusion rates in randomized controlled trials of treatments for physical conditions: a systematic review. Trials. 2020;21(1):1–11. 32102686 10.1186/s13063-020-4139-0 PMC7045589

[9] Hanlon P, Hannigan L, Rodriguez-Perez J, Fischbacher C, Welton NJ, Dias S, et al. Representation of people with comorbidity and multimorbidity in clinical trials of novel drug therapies: an individual-level participant data analysis. BMC Med. 2019;17(1):201. 31711480 10.1186/s12916-019-1427-1 PMC6849229

[10] Hwang K, Moore KJ, Chong TW, Williams S, Batchelor F. Improving clinical practice guidelines for older people: considerations and recommendations for more inclusive and ageing-relevant guidelines. Lancet Healthy Longev. 2022;3(5):e316-7. 36098306 10.1016/S2666-7568(22)00074-5

[11] Hanlon P, Butterly E, Shah ASV, Hannigan LJ, Wild SH, Guthrie B, Mair FS, Dias S, Welton NJ, McAllister DA. Assessing trial representativeness using serious adverse events: an observational analysis using aggregate and individual-level data from clinical trials and routine healthcare data. BMC Med. 2022;20(1):410. 10.1186/s12916-022-02594-9 PMC9615407 36303169

[12] Hanlon P, Corcoran N, Rughani G, Shah AS, Mair FS, Guthrie B, et al. Observed and expected serious adverse event rates in randomised clinical trials for hypertension: an observational study comparing trials that do and do not focus on older people. Lancet Healthy Longev. 2021;2(7):e398-406. 34240062 10.1016/S2666-7568(21)00092-1 PMC8245327

[13] Chiang JI, Hanlon P, Li T-C, Jani BD, Manski-Nankervis J-A, Furler J, et al. Multimorbidity, mortality, and HbA1c in type 2 diabetes: a cohort study with UK and Taiwanese cohorts. PLoS Med. 2020;17(5):e1003094. 32379755 10.1371/journal.pmed.1003094 PMC7205223

[14] Chiang JI, Jani BD, Mair FS, Nicholl BI, Furler J, O’Neal D, et al. Associations between multimorbidity, all-cause mortality and glycaemia in people with type 2 diabetes: a systematic review. PLoS One. 2018;13(12):e0209585. 30586451 10.1371/journal.pone.0209585 PMC6306267

[15] Witham MD, Stott DJ. Conducting and reporting trials for older people. Age Ageing. 2017;46(6):889–94. 28985243 10.1093/ageing/afx153

[16] What is a serious adverse event? https://www.fda.gov/safety/reporting-serious-problems-fda/what-serious-adverse-event. Accessed Sep 2025.

[17] Hanlon P, Butterly E, Wei L, Wightman H, Almazam SAM, Alsallumi K, et al. Age and sex differences in efficacy of treatments for type 2 diabetes: a network meta-analysis. JAMA. 2025;333(12):1062–73. 39899304 10.1001/jama.2024.27402 PMC11791772

[18] Jones KH, Ford DV, Thompson S, Lyons R. A profile of the Sail Databank on the UK secure research platform. Int J Popul Data Sci. 2019;4(2):1134. 10.23889/ijpds.v4i2.1134 PMC8142954 34095541

[19] Lewis JD, Bilker WB, Weinstein RB, Strom BL. The relationship between time since registration and measured incidence rates in the general practice research database. Pharmacoepidemiol Drug Saf. 2005;14(7):443–51. 15898131 10.1002/pds.1115

[20] Ho IS, Azcoaga-Lorenzo A, Akbari A, Davies J, Khunti K, Kadam UT, Lyons RA, McCowan C, Mercer SW, Nirantharakumar K. Measuring multimorbidity in research: Delphi consensus study. BMJ Med. 2022;1(1):e000247. 10.1136/bmjmed-2022-000247 PMC9978673 36936594

[21] Levey AS, Stevens LA, Schmid CH, Zhang Y, Castro AF III, Feldman HI, et al. A new equation to estimate glomerular filtration rate. Ann Intern Med. 2009;150(9):604–12. 19414839 10.7326/0003-4819-150-9-200905050-00006 PMC2763564

[22] MacRae C, Morales D, Mercer SW, Lone N, Lawson A, Jefferson E, et al. Impact of data source choice on multimorbidity measurement: a comparison study of 2.3 million individuals in the Welsh National Health Service. BMC Med. 2023;21(1):309. 37582755 10.1186/s12916-023-02970-z PMC10426056

[23] Vonesh E, Tighiouart H, Ying J, Heerspink HL, Lewis J, Staplin N, et al. Mixed-effects models for slope-based endpoints in clinical trials of chronic kidney disease. Stat Med. 2019;38(22):4218–39. 31338848 10.1002/sim.8282

[24] Inker LA, Collier W, Greene T, Miao S, Chaudhari J, Appel GB, et al. A meta-analysis of GFR slope as a surrogate endpoint for kidney failure. Nat Med. 2023;29(7):1867–76. 37330614 10.1038/s41591-023-02418-0 PMC13037386

[25] Inker LA, Heerspink HJL, Tighiouart H, Levey AS, Coresh J, Gansevoort RT, et al. GFR slope as a surrogate end point for kidney disease progression in clinical trials: a meta-analysis of treatment effects of randomized controlled trials. J Am Soc Nephrol. 2019; 30(9):1735–45. 10.1681/ASN.2019010007 PMC6727261 31292197

[26] Wightman H, Butterly E, Wei L, McChrystal R, Sattar N, Adler A, et al. Frailty in randomized controlled trials of glucose-lowering therapies for type 2 diabetes: An individual participant data meta-analysis of frailty prevalence, treatment efficacy, and adverse events. Plos Med. 2025;22(4):e1004553. 40193407 10.1371/journal.pmed.1004553 PMC12052138

[27] Malik ME, Butt JH, Strange JE, Falkentoft AC, Jensen J, Andersson C, et al. Initiation of SGLT2 inhibitors and GLP-1 receptor agonists according to level of frailty in people with type 2 diabetes and cardiovascular disease in Denmark: a cross-sectional, nationwide study. The Lancet Healthy Longevity. 2023;4(10):e552–60. 37734395 10.1016/S2666-7568(23)00164-2

[28] Bousetta R, McAllister DA, Wightman H, Lewsey J, Hanlon P. Does the frailty index applied to randomised controlled trials really measure frailty? Age and Ageing. 2025;54(10);afaf314. 10.1093/ageing/afaf314 PMC12551376 41134536

