# Supplementary material for: Assessing the representativeness of trials of Sodium-glucose Cotransporter-2 inhibitors in type 2 diabetes: a comparison of individual-level trial data and people newly prescribed treatment in a Welsh routine care database
Source: BMC Med. 2025 Nov 26;23:661. doi: 10.1186/s12916-025-04492-2 (PMC12659586; doi:10.1186/s12916-025-04492-2)

Supplementary appendix

Table S1: Age and sex characteristics for community SGLT2i treated individuals who meet eligibility criteria for each trial, those who did not meet eligibility criteria, and actual trial participants.

| Trial | Total n | | | Mean age (sd) | | | Total female (%) | | |
| --- | --- | --- | --- | --- | --- | --- | --- | --- | --- |
|  | Ineligible treated (community) | Eligible treated (community) | Trial participants | Ineligible treated (community) | Eligible treated (community) | Trial participants | Ineligible treated (community) | Eligible treated (community) | Trial participants |
| EMPA-REG OUTCOME | 26338 | 3206 | 7063 | 59.6 (11.7) | 66.6 (9.7) | 63.1 (8.6) | 11300 (43%) | 927 (29%) | 2015 (29%) |
| CANVAS-R | 20046 | 9498 | 5811 | 58.6 (11.8) | 64 (10.5) | 62.4 (8.4) | 8951 (45%) | 3276 (34%) | 2163 (37%) |
| CANVAS | 20046 | 9498 | 4330 | 58.6 (11.8) | 64 (10.5) | 60.9 (8.1) | 8951 (45%) | 3276 (34%) | 1469 (34%) |
| CREDENCE | 28896 | 648 | 4401 | 60.3 (11.7) | 63.6 (11.4) | 55.9 (10.1) | 12040 (42%) | 187 (29%) | 1494 (34%) |
| EMPA-REG RENAL | 22718 | 6826 | 741 | 58.5 (11.7) | 66.6 (9.4) | 63.9 (8.8) | 9443 (42%) | 2784 (41%) | 309 (42%) |
| NCT02182830 (Hypertension) | 20518 | 9026 | 157 | 59.7 (12.2) | 61.8 (10.2) | 56.9 (9.7) | 8682 (42%) | 3545 (39%) | 76 (48%) |
| NCT01106651 (Older people) | 18337 | 15593 | 716 | 57 (12.9) | 66.1 (6.3) | 63.6 (6.3) | 7942 (43%) | 5864 (38%) | 320 (45%) |
| EMPA-REG MDI | 27679 | 1865 | 545 | 60.3 (11.7) | 61.3 (12) | 57 (9.4) | 11411 (41%) | 816 (44%) | 297 (54%) |
| EMPA-REG BP | 18696 | 10848 | 845 | 58.8 (12.1) | 63 (10.4) | 60.3 (9) | 7867 (42%) | 4360 (40%) | 339 (40%) |
| CANTATA-D2 | 23464 | 6080 | 756 | 60 (11.9) | 62 (10.8) | 56.6 (9.5) | 9957 (42%) | 2270 (37%) | 334 (44%) |
| CANTATA-MSU | 23352 | 6192 | 469 | 60.1 (12.1) | 61.3 (10.2) | 56.7 (9.3) | 9915 (42%) | 2312 (37%) | 230 (49%) |
| CANTATA-MP | 28848 | 696 | 343 | 60.3 (11.7) | 61.6 (9.5) | 57.1 (10.1) | 11995 (42%) | 232 (33%) | 126 (37%) |
| NCT01734785 | 20012 | 9532 | 1536 | 60.5 (12.1) | 60.1 (10.9) | 55.3 (9.6) | 8759 (44%) | 3468 (36%) | 623 (41%) |
| CANTATA-D | 11894 | 17650 | 1284 | 61.2 (13) | 59.8 (10.7) | 53.8 (9.5) | 5135 (43%) | 7092 (40%) | 679 (53%) |
| EMPA-REG H2H-SU | 16464 | 13080 | 1380 | 60.2 (12.1) | 60.6 (11.1) | 56.7 (10.2) | 7229 (44%) | 4998 (38%) | 629 (46%) |
| NCT01809327 | 28189 | 1355 | 1186 | 60.5 (11.7) | 57.8 (10.9) | 51.9 (10.1) | 11616 (41%) | 611 (45%) | 617 (52%) |
| NCT01368081 | 11838 | 17706 | 1189 | 59.3 (11.9) | 61.1 (11.5) | 60.4 (10.4) | 4899 (41%) | 7328 (41%) | 338 (28%) |
| NCT01719003 | 28637 | 907 | 1166 | 60.4 (11.7) | 59.3 (12.5) | 53.3 (10.8) | 11838 (41%) | 389 (43%) | 525 (45%) |
| CANTA-M | 7023 | 22521 | 586 | 67.1 (13.9) | 58.3 (10.1) | 55.2 (10.6) | 2975 (42%) | 9252 (41%) | 327 (56%) |
| EMPA-REG METSU | 16685 | 12859 | 1504 | 61.1 (12.1) | 59.5 (11.2) | 55.9 (9.9) | 7201 (43%) | 5026 (39%) | 688 (46%) |
| EMPA-REG EXTEND PIO | 29046 | 498 | 499 | 60.3 (11.7) | 62.6 (9.9) | 54.5 (9.8) | 12061 (42%) | 166 (33%) | 258 (52%) |
| NCT02453555 | 18662 | 10882 | 1179 | 60.2 (12.1) | 60.7 (10.9) | 59.9 (10.3) | 8333 (45%) | 3894 (36%) | 268 (23%) |
| NCT02489968 | 28513 | 1031 | 2046 | 60.3 (11.7) | 61.4 (12.6) | 57.7 (9.9) | 11757 (41%) | 470 (46%) | 549 (27%) |
| EMPA-REG MONO | 28725 | 819 | 985 | 60.4 (11.7) | 60.6 (12.2) | 54.5 (11.1) | 11855 (41%) | 372 (45%) | 370 (38%) |
| NCT01381900 | 11894 | 17650 | 678 | 61.2 (13) | 59.8 (10.7) | 49.2 (10.1) | 5135 (43%) | 7092 (40%) | 314 (46%) |

Table S2: Mean (sd) number of comorbidities among community SGLT2i treated individuals who meet eligibility criteria for each trial, those who did not meet eligibility criteria, and actual trial participants.

| Trial | Total comorbidities: Mean (sd) | | | Cardiometabolic comorbidities: Mean (sd) | | | Noncardiometabolic comorbidities: Mean (sd) | | |
| --- | --- | --- | --- | --- | --- | --- | --- | --- | --- |
|  | Ineligible treated (community) | Eligible treated (community) | Trial participants | Ineligible treated (community) | Eligible treated (community) | Trial participants | Ineligible treated (community) | Eligible treated (community) | Trial participants |
| EMPA-REG OUTCOME | 2.9 (2.1) | 4.4 (2.1) | 3.4 (1.6) | 0.9 (0.9) | 2.2 (1) | 2.4 (0.9) | 2.1 (1.8) | 2.2 (1.7) | 0.9 (0.9) |
| CANVAS-R | 2.8 (2.1) | 3.7 (2.3) | 2.9 (1.8) | 0.8 (0.9) | 1.5 (1.1) | 1.9 (1.1) | 2 (1.7) | 2.3 (1.8) | 1 (1) |
| CANVAS | 2.8 (2.1) | 3.7 (2.3) | 2.7 (1.7) | 0.8 (0.9) | 1.5 (1.1) | 1.8 (1) | 2 (1.7) | 2.3 (1.8) | 0.9 (0.9) |
| CREDENCE | 3.1 (2.2) | 3.7 (2.3) | 2.7 (1.8) | 1 (1) | 1.5 (1.2) | 1.6 (1) | 2.1 (1.8) | 2.2 (1.8) | 1 (1) |
| EMPA-REG RENAL | 3.1 (2.2) | 3.1 (2.1) | 2.3 (1.5) | 0.9 (1) | 1.2 (1) | 1.4 (0.9) | 2.2 (1.8) | 1.9 (1.6) | 0.9 (0.9) |
| NCT02182830 (Hypertension) | 3.1 (2.3) | 3.1 (1.8) | 2.3 (1.4) | 0.8 (1.1) | 1.4 (0.7) | 1.2 (0.6) | 2.3 (1.8) | 1.7 (1.5) | 1 (1) |
| NCT01106651 (Older people) | 3 (2.2) | 3.2 (2.1) | 2.2 (1.5) | 0.9 (1) | 1.1 (1) | 1.1 (0.7) | 2.1 (1.8) | 2.1 (1.7) | 1.1 (1.1) |
| EMPA-REG MDI | 3.1 (2.1) | 3.7 (2.4) | 2.1 (1.6) | 1 (1) | 1.2 (1.2) | 1.3 (1) | 2.1 (1.7) | 2.5 (1.9) | 0.8 (0.8) |
| EMPA-REG BP | 2.8 (2.2) | 3.6 (2.1) | 1.9 (1) | 0.7 (1) | 1.5 (0.8) | 1.3 (0.5) | 2.1 (1.8) | 2.2 (1.8) | 0.7 (0.7) |
| CANTATA-D2 | 3.1 (2.2) | 3 (2.1) | 1.8 (1.5) | 1 (1) | 1 (1) | 1 (0.8) | 2.1 (1.8) | 2 (1.7) | 0.8 (0.8) |
| CANTATA-MSU | 3.1 (2.2) | 3 (2.1) | 1.8 (1.6) | 1 (1) | 1 (0.9) | 0.9 (0.8) | 2.1 (1.8) | 2 (1.7) | 0.8 (0.8) |
| CANTATA-MP | 3.1 (2.2) | 2.7 (1.9) | 1.7 (1.4) | 1 (1) | 0.8 (0.7) | 0.9 (0.7) | 2.1 (1.8) | 1.9 (1.6) | 0.9 (0.9) |
| NCT01734785 | 3.3 (2.2) | 2.7 (2) | 1.6 (1.3) | 1 (1.1) | 0.9 (0.9) | 0.9 (0.8) | 2.2 (1.8) | 1.8 (1.6) | 0.8 (0.8) |
| CANTATA-D | 3.3 (2.3) | 3 (2.1) | 1.5 (1.4) | 1.1 (1.1) | 0.9 (0.9) | 0.9 (0.9) | 2.2 (1.8) | 2 (1.7) | 0.6 (0.6) |
| EMPA-REG H2H-SU | 3.5 (2.3) | 2.6 (1.9) | 1.4 (1.2) | 1 (1.1) | 0.9 (0.9) | 0.8 (0.7) | 2.4 (1.8) | 1.7 (1.6) | 0.6 (0.6) |
| NCT01809327 | 3.1 (2.2) | 3 (2.1) | 1.4 (1.4) | 1 (1) | 0.9 (0.9) | 0.9 (0.9) | 2.1 (1.8) | 2.1 (1.8) | 0.5 (0.5) |
| NCT01368081 | 3.2 (2.3) | 3 (2.1) | 1.4 (1.2) | 1 (1.1) | 1 (1) | 0.7 (0.7) | 2.2 (1.8) | 2 (1.7) | 0.6 (0.6) |
| NCT01719003 | 3.1 (2.2) | 2.6 (1.9) | 1.4 (1.4) | 1 (1) | 0.9 (1) | 0.7 (0.8) | 2.1 (1.8) | 1.7 (1.5) | 0.6 (0.6) |
| CANTA-M | 3.7 (2.4) | 2.9 (2.1) | 1.3 (1.2) | 1.3 (1.2) | 0.9 (0.9) | 0.7 (0.7) | 2.3 (1.8) | 2 (1.7) | 0.6 (0.6) |
| EMPA-REG METSU | 3.5 (2.3) | 2.5 (1.8) | 1.3 (1.3) | 1.1 (1.1) | 0.9 (0.9) | 0.8 (0.8) | 2.5 (1.8) | 1.6 (1.5) | 0.5 (0.5) |
| EMPA-REG EXTEND PIO | 3.1 (2.2) | 2.4 (1.8) | 1.3 (1.3) | 1 (1) | 0.8 (0.8) | 0.8 (0.9) | 2.1 (1.8) | 1.6 (1.5) | 0.4 (0.4) |
| NCT02453555 | 3.4 (2.2) | 2.5 (1.9) | 1.2 (1) | 1.1 (1.1) | 0.9 (0.9) | 0.6 (0.6) | 2.4 (1.8) | 1.6 (1.5) | 0.6 (0.6) |
| NCT02489968 | 3.1 (2.2) | 3.1 (2) | 1.2 (1) | 1 (1) | 1 (1) | 0.6 (0.6) | 2.1 (1.8) | 2.1 (1.7) | 0.5 (0.5) |
| EMPA-REG MONO | 3.1 (2.2) | 2.8 (2) | 1.2 (1.2) | 1 (1) | 1 (1) | 0.6 (0.7) | 2.1 (1.8) | 1.8 (1.6) | 0.5 (0.5) |
| NCT01381900 | 3.3 (2.3) | 3 (2.1) | 0.6 (0.7) | 1.1 (1.1) | 0.9 (0.9) | 0.5 (0.6) | 2.2 (1.8) | 2 (1.7) | 0.1 (0.1) |

Table S3: Ratio of comorbidity counts comparing (1) trial participants and community SGLT2i treated people who were eligible for the trial, and (2) community SGLT2i treated people who were eligible for the trial and community SGLT2i treated people who were not eligible for the trial

| Trial | Ratio of total comorbidities | | Ratio of cardiometabolic comorbidities | | Ratio of non-cardiometabolic comorbidities | |
| --- | --- | --- | --- | --- | --- | --- |
|  | Trial participants :  Eligible and treated | Eligible and treated :  ineligible and treated | Trial participants :  Eligible and treated | Eligible and treated :  ineligible and treated | Trial participants :  Eligible and treated | Eligible and treated :  ineligible and treated |
| EMPA-REG OUTCOME | 0.77 | 1.49 | 1.13 | 2.52 | 0.41 | 1.06 |
| CANVAS-R | 0.78 | 1.32 | 1.31 | 1.86 | 0.44 | 1.11 |
| CANVAS | 0.73 | 1.32 | 1.23 | 1.86 | 0.42 | 1.11 |
| CREDENCE | 0.72 | 1.21 | 1.07 | 1.56 | 0.47 | 1.05 |
| EMPA-REG RENAL | 0.76 | 0.98 | 1.22 | 1.24 | 0.47 | 0.86 |
| NCT02182830 (Hypertension) | 0.73 | 1.01 | 0.89 | 1.67 | 0.6 | 0.77 |
| NCT01106651 (Older people) | 0.69 | 1.07 | 1 | 1.19 | 0.53 | 1.02 |
| EMPA-REG MDI | 0.57 | 1.2 | 1.07 | 1.23 | 0.33 | 1.19 |
| EMPA-REG BP | 0.52 | 1.31 | 0.85 | 2.05 | 0.3 | 1.05 |
| CANTATA-D2 | 0.58 | 0.97 | 0.95 | 1.05 | 0.39 | 0.94 |
| CANTATA-MSU | 0.59 | 0.95 | 0.91 | 1.01 | 0.42 | 0.93 |
| CANTATA-MP | 0.64 | 0.86 | 1.04 | 0.83 | 0.46 | 0.88 |
| NCT01734785 | 0.61 | 0.82 | 1.01 | 0.85 | 0.42 | 0.8 |
| CANTATA-D | 0.5 | 0.9 | 0.94 | 0.87 | 0.3 | 0.92 |
| EMPA-REG H2H-SU | 0.55 | 0.76 | 0.91 | 0.89 | 0.35 | 0.71 |
| NCT01809327 | 0.48 | 0.96 | 1.06 | 0.86 | 0.24 | 1.01 |
| NCT01368081 | 0.46 | 0.93 | 0.76 | 0.96 | 0.32 | 0.91 |
| NCT01719003 | 0.52 | 0.83 | 0.83 | 0.89 | 0.37 | 0.81 |
| CANTA-M | 0.45 | 0.8 | 0.81 | 0.67 | 0.29 | 0.86 |
| EMPA-REG METSU | 0.51 | 0.72 | 0.87 | 0.83 | 0.32 | 0.67 |
| EMPA-REG EXTEND PIO | 0.53 | 0.77 | 1.01 | 0.83 | 0.28 | 0.75 |
| NCT02453555 | 0.49 | 0.74 | 0.68 | 0.84 | 0.38 | 0.69 |
| NCT02489968 | 0.38 | 0.99 | 0.66 | 0.97 | 0.26 | 0.99 |
| EMPA-REG MONO | 0.42 | 0.89 | 0.65 | 0.96 | 0.29 | 0.85 |
| NCT01381900 | 0.2 | 0.9 | 0.49 | 0.87 | 0.07 | 0.92 |

Figure S1 – distribution of total, cardiometabolic and non-cardiometabolic comorbidities


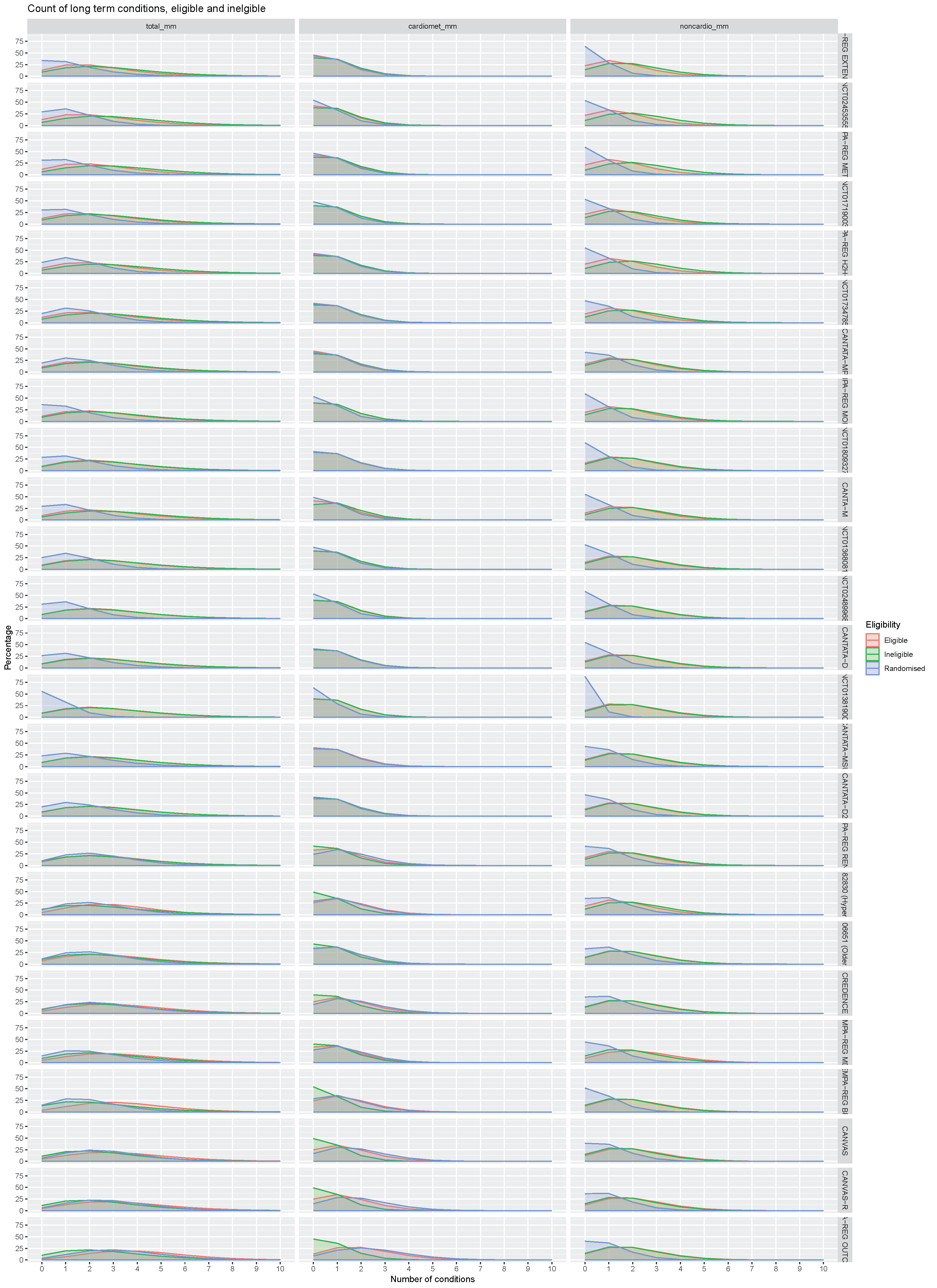

Supplement: Supplementary file 1 — Additional file 1: Table S1. Age and sex characteristics for community SGLT2i treated individuals who meet eligibility criteria for each trial, those who did not meet eligibility criteria, and actual trial participants. Table S2. Mean (sd) number of comorbidities among community SGLT2i treated individuals who meet eligibility criteria for each trial, those who did not meet eligibility criteria, and actual trial participants. Table S3. Ratio of comorbidity counts comparing (1) trial participants and community SGLT2i treated people who were eligible for the trial, and (2) community SGLT2i treated people who were eligible for the trial and community SGLT2i treated people who were not eligible for the trial. Figure S1. Distribution of total, cardiometabolic and non-cardiometabolic comorbidities in trials and in routine care. [file 12916_2025_4492_MOESM1_ESM.docx]
